# Low Acceptability of A/H1N1 Pandemic Vaccination in French Adult Population: Did Public Health Policy Fuel Public Dissonance?

**DOI:** 10.1371/journal.pone.0010199

**Published:** 2010-04-16

**Authors:** Michaël Schwarzinger, Rémi Flicoteaux, Sébastien Cortarenoda, Yolande Obadia, Jean-Paul Moatti

**Affiliations:** 1 INSERM, U912: Economic and Social Sciences, Health Systems and Societies, Marseille, France; 2 University Aix Marseille, IRD, UMR-S912, Marseille, France; 3 ORS PACA, Southeastern Health Regional Observatory, Marseille, France; 4 University Diderot Paris 7, Laboratoire de pathologies infectieuses, Paris, France; Duke University Medical Center, United States of America

## Abstract

**Background:**

In July 2009, French public health authorities embarked in a mass vaccination campaign against A/H1N1 2009 pandemic-influenza. We explored the attitudes and behaviors of the general population toward pandemic vaccination.

**Methodology/Principal Findings:**

We conducted a cross-sectional online survey among 2,253 French representative adults aged 18 to 64 from November 17 to 25, 2009 (completion rate: 93.8%). The main outcome was the acceptability of A/H1N1 vaccination as defined by previous receipt or intention to get vaccinated (“Yes, certainly”, “Yes, probably”). Overall 17.0% (CI 95%, 15.5% to 18.7%) of respondents accepted A/H1N1 vaccination. Independent factors associated with acceptability included: male sex (p = .0001); older age (p = .002); highest or lowest level of education (p = .016); non-clerical occupation (p = .011); having only one child (p = .008); and having received seasonal flu vaccination in prior 3 years (p<.0001). Acceptability was also significantly higher among pregnant women (37.9%) and other at risk groups with chronic diseases (34.8%) (p = .002). Only 35.5% of respondents perceived A/H1N1 influenza illness as a severe disease and 12.7% had experienced A/H1N1 cases in their close relationships with higher acceptability (p<.0001 and p = .006, respectively). In comparison to 26.0% respondents who did not consult their primary care physician, acceptability was significantly higher among 8.0% respondents who were formally advised to get vaccinated, and lower among 63.7% respondents who were not advised to get vaccinated (respectively: 15.8%, 59.5% and 11.7%- p<.0001). Among respondents who refused vaccination, 71.2% expressed concerns about vaccine safety.

**Conclusions/Significance:**

Our survey occurred one week before the peak of the pandemic in France. We found that alarming public health messages aiming at increasing the perception of risk severity were counteracted by daily personal experience which did not confirm the threat, while vaccine safety was a major issue. This dissonance may have been amplified by having not involved primary care physicians in the mass vaccination campaign.

## Introduction

Following the recommendations of the World Health Organization [1], French public health authorities have decided to embark in a mass vaccination campaign to mitigate the transmission of the A/H1N1 2009 pandemic-influenza. In July 2009, the French government bought a total of 94 million doses of vaccines with the explicit goal to provide two successive vaccine doses to 75% of the whole population (62.5 million inhabitants in metropolitan France). Such a goal was quite ambitious as compared to usual rates of seasonal flu vaccine uptake in the population: 50% in targeted subgroups at risk for influenza complications; and less than 25% otherwise [2], [3]. Another key decision of French authorities was to implement the A/H1N1 immunization campaign in mass vaccination centers, especially put in place on this occasion, in contrast to the usual prescription and administration of seasonal flu vaccines by general practitioners and other groups of ambulatory specialist physicians (mainly, pediatricians and gynecologists). In particular, primary care physicians were not associated with the A/H1N1 immunization campaign for economic and logistical reasons [4].

On October 20, 2009, the distribution of available vaccine supply started in hospitals for 1.2 million health care professionals including doctors and nurses of primary care settings [5]. On November 12, the access to vaccines was extended in mass vaccination centers for: 1.7 million household contacts and caregivers for children younger than 6 months of age; 880,000 additional health care professionals in primary care settings; and 2.8 million individuals aged 6 months to 64 years at risk for A/H1N1 influenza complications (including pregnant women and individuals with chronic pulmonary disease, chronic heart disease or diabetes identified as “priority groups” by the Advisory Public Health Council) [5]. On December 1, the vaccination campaign was extended to other at-risk individuals older than 65 years, while vaccination began in schools. All targeted individuals were identified by the French Sickness Insurance Fund (Social Security), and received a personalized invitation letter from the Minister of Health that was necessary to access the closest vaccination center.

It is a well-established fact that risk perceptions influence influenza vaccine uptake [6], [7], and that there is a need to consider and understand factors underlying people's decision about vaccination to create an effective immunization program [8], [9], [10]. The French National Research Institute for Microbiology and Infectious Diseases (IMMI), that is part of the French National Institutes of Health (INSERM and the other involved health research agencies), has therefore established, through the Web, a representative panel of the French population aged 18 to 64 years in order to follow the evolution of attitudes and behaviors toward the mass vaccination campaign. The first cross-sectional survey was carried out in the panel from November 17 to 25, 2009, to assess the acceptability of A/H1N1 pandemic vaccination in the French general population, as well as its main determinants including risk perception, at the initiation of the campaign. Since seasonal flu immunization behaviors in the general population have been shown to be associated with behaviors, attitudes, and advice from primary care physicians [2], [11], [12], [13], we also assessed whether the choice not to mobilize primary care physicians may negatively affect compliance with the mass vaccination campaign.

## Methods

### Ethics statement

The survey was approved by the National Data Protection Authority (Commission Nationale Informatique et Libertés/CNIL) which is in charge of ethical issues and protection of individual data collection in France, and written informed consent was obtained from each participant.

### Sampling procedure

A sample was randomly selected from an online research panel of more than 220,000 nationally representative households of the French general population developed and maintained by IPSOS Interactive Services (Gentilly, France), a survey research firm (http://www.ipsos-interactive.com/). The sample size of 2,200 was calculated to obtain a maximum margin of sampling error of ±2.0 percentage points for an overall acceptability of A/H1N1 vaccination of 50%. A total of 19,780 households were randomly drawn to reach the sample size within a week. Prior information on the panelists was used to determine eligibility and to draw a stratified random sample with oversampling of panelists with low response rates. To be eligible, panelists had to be aged 18 to 64 years and having not answered a survey on communicable diseases in the last twelve weeks or more than 6 surveys in the last four weeks. To limit coverage bias, random sampling was stratified to match French official census statistics for gender; age (18–24; 25–34; 35–44; 45–54; 55–64); occupation (5 categories); household size (1; 2; 3; 4; 5 members or more), population in the area of residence (less than 20,000; 20,000 to 100,000; 100,000 to 200,000; 200,000 inhabitants or more); and region (Ile-de-France including Paris; North-East; North-West; South-East; South-West). To limit selection bias, panelists with low response rates were oversampled relative to others, e.g. fifty panelists with a 1% chance to take the survey were randomly drawn for one panelist with a 50% chance. In addition, panelists were invited by email to participate to an “academic survey” dealing “with protective behaviors against communicable diseases”. This initial invitation did not refer explicitly to the influenza-pandemic and did not mention specifically the words “vaccination”, “swine flu”, or “pandemic”. Finally, 2,093 (12.0%) panelists had completed the survey out of the 17,425 invitations mailed out on November 17, and an additional 2,355 households oversampling young single males were invited on November 23 to achieve a French representative sample.

### Survey instrument

The online questionnaire used an adaptative questioning to reduce the number of questions with one question per screen [14]. The online questionnaire is available in French language at http://www.enquetegrippeH1N1.org with an English translation at http://www.H1N1flusurvey.org. Nine questions dealt with the socio-demographic characteristics of the respondent. Two questions, validated in previous French national health surveys [15], assessed individual's subjective health state using a 5-point scale (from “poor” to “excellent”), and consultations with physicians in the prior six months. In addition, respondents were asked if they were pregnant, if they had a chronic disease, and what was their level of compliance with seasonal flu vaccination and other vaccinations recommended by their primary care physician (for example, before travelling abroad).

Respondents were then asked if they had an episode of flu since May 2009, and two questions allowed to determine the extent to which these episodes could be related to the A/H1N1 2009 influenza virus (as confirmed by a lab test in ambulatory medicine or by a hospitalization). Three additional questions asked whether respondents know personally someone who contracted A/H1N1 flu (family members, work colleagues, neighborhood and community).

Behaviors and attitudes toward A/H1N1 vaccination were assessed using three successive questions. First, respondents were asked if they had already been vaccinated (yes/no). All those who had not been vaccinated were subsequently asked if they were willing to get vaccinated using a 4-point scale (“Yes, certainly”, “Yes, probably”, “No, probably not”, “No, certainly not”). Finally, the main reasons for getting vaccinated or not were asked with two alternative multiple choices questions [16].

Four questions dealt with respondents' risk perceptions of the threat associated with the A/H1N1 influenza-pandemic: two questions asked respondents whether they were “not at all worried”, “somewhat worried”, “worried” or “very worried” about the A/H1N1 influenza-pandemic for themselves or their close relatives; another question asked if the respondent personally felt at “higher risk”, “same risk”, “lower risk than average” or “not at all at risk” to contract A/H1N1 influenza infection; finally, respondents were asked if they estimated that A/H1N1 influenza-pandemic illness should be considered a “severe disease” (“not at all severe”, “somewhat severe”, “severe”, “very severe”).

Order response bias from subjective assessment was controlled by random allocation of: the direction of all ordinal scales like the ones just mentioned; and the two sections of questions addressing attitudes and behaviors on the one hand, and risk perceptions on the other hand. In addition, participants had unlimited time to complete the survey.

Finally, we used external data collected regularly on the panelists and made available for the present analysis by courtesy of IPSOS Interactive Services. IPSOS panel data were used to cross-validate the 6 stratification variables as well as self-declared health status, including pregnancy and the presence of chronic diseases.

### Statistical analysis

The main outcome was the acceptability of A/H1N1 vaccination as defined by previous receipt or intention to get vaccinated (“Yes, certainly”, “Yes, probably”) versus unwillingness to get vaccinated (“No, probably not”, “No, certainly not”). Univariate analyses were carried out using chi-square tests. For ordinal variables, the Cochran-Armitage test for trend was performed. Multivariate logistic regression was carried out with acceptability of A/H1N1 vaccination as the dependent variable. All variables significant at p<.15 in univariate analyses were introduced in the initial multivariate model. If several variables were strongly linked, then all could be considered as “proxies” of the same phenomenon (i.e. risk perception, level of compliance with vaccination), and a single variable was selected to avoid problems of multicollinearity. All covariates were selected using a backward selection (p<.05 to stay). However, sample stratification variables (gender, age, occupation, household size, population in the area of location and region) were forced in the final model even if they did not meet the p<.05 criterion. Finally, we looked for additional two-way interaction effects using a backward selection (p<.05 to stay) on the final model augmented with all two-way interaction effects. All analyses were based on two-sided p values, with p<.05 considered to indicate statistical significance. All analyses were carried out using SAS 9.1.3 statistical software (SAS Institute, Cary, NC).

## Results

A total of 2,253 adults aged 18 to 64 completed the online survey between November 17 and 25, 2009 (completion rate  = 93.8%). No differences for the six socio-demographic and geographic variables used for stratification were found between respondents and the French general population as observed in the latest census statistics [17]. Eighty-six (3.8%) respondents declared that they had an episode of flu in the prior three months and were excluded from the present analysis, although 61 (71%) of these respondents did not receive a formal diagnosis of an infection by A/H1N1 2009 influenza virus.

Overall, the acceptability of A/H1N1 vaccination was 17.0% (CI 95%, 15.5% to 18.7%). Only a minority (1.9% -n = 42) had already received the A/H1N1 pandemic vaccine (Table 1). This proportion was higher (10.9% -n = 13) among health care professionals in accordance with the timing of the vaccination campaign. An additional number of 327 respondents (15.1% in total sample, 9.2% among health care professionals) declared that they had the intention to get vaccinated. The most frequent quoted reason to accept vaccination was “self-protection” (74.5%), while only one quarter (24.1%) invoked that getting vaccinated was “a civic duty” (Table 2). Among 1,798 respondents who did not accept vaccination, the main reasons were concerns about vaccine safety and fear of vaccine side effects (respectively quoted by 71.2% and 68.4%).

**Table 1:** Acceptability of A/H1N1 pandemic vaccination in French adult population (18-64 years) and its determinants (online survey, November 17 to 25, 2009, N = 2,167).

	Acceptance of A/H1N1 pandemic vaccination, N (%)						Total respondents (N = 2,167)		Univariate comparison (1)+(2) vs. (3)	Multivariate logistic model	
	Yes, N = 369 (17.0%)				No, N = 1,798 (83.0%)					(1)+(2) vs. (3)	
	(1) Already vaccinated, N = 42 (1.9%)		(2) Intention to get vaccinated, N = 327 (15.1%)		(3) No intention to get vaccinated				p-value†	Adjusted OR [CI 95%]‡	p-value‡
**Sex ** #									<.0001		.0001
Male	29	(2.6)	201	(18.3)	872	(79.1)	1,102	(50.9)		Ref.	
Female	13	(1.2)	126	(11.8)	926	(87.0)	1,065	(49.1)		0.57 [0.43; 0.76]	
**Age** #									<.0001		.002
18-34	10	(1.2)	96	(11.5)	728	(87.3)	834	(38.5)		Ref.	
35-54	19	(1.9)	163	(16.3)	820	(81.8)	1,002	(46.2)		1.41 [1.03; 1.93]	
≥55	13	(3.9)	68	(20.6)	250	(75.5)	331	(15.3)		2.11 [1.38; 3.24]	
**Education** #									.001		.016
University graduates	8	(3.5)	50	(21.8)	171	(74.7)	229	(10.6)		Ref.	
High school graduates or college undergraduates	19	(1.5)	163	(12.8)	1,094	(85.7)	1,276	(58.9)		0.53 [0.34; 0.82]	
Some high school	14	(2.4)	100	(16.8)	480	(80.8)	594	(27.4)		0.69 [0.42; 1.13]	
Primary level of education	1	(1.5 )	14	(20.6)	53	(77.9)	68	(3.1)		0.87 [0.40; 1.92]	
**Social Grade** #									.005		.011
Clerical	12	(2.0)	62	(10.4)	521	(87.6)	595	(27.5)		Ref.	
Managerial	5	(1.8)	56	(20.1)	218	(78.1)	279	(12.9)		2.14 [1.34; 3.41]	
Manual	7	(1.3)	85	(16.2)	434	(82.5)	526	(24.3)		1.60 [1.07; 2.37]	
Self Employed	1	(0.8)	22	(19.0)	93	(80.2)	116	(5.3)		2.18 [1.19; 3.99]	
Retired / Unemployed	17	(2.6)	102	(15.7)	532	(81.7)	651	(30.0)		1.49 [1.01; 2.20]	
**Number of adults in household** #									.84		.85
One	10	(1.7)	85	(14.6)	489	(83.7)	584	(26.9)		Ref.	
Two	23	(2.0)	174	(15.3)	938	(82.7)	1,135	(52.4)		1.00 [0.72; 1.37]	
More than two	9	(2.0)	68	(15.2)	371	(82.8)	448	(20.7)		1.10[0.74; 1.63]	
**Number of children in household** #									.008		.008
None	20	(1.7)	152	(13.4)	965	(84.9)	1,137	(52.5)		Ref.	
One	15	(3.2)	85	(18.3)	364	(78.5)	464	(21.4)		1.68 [1.21; 2.35]	
More than one	7	(1.2)	90	(15.9)	469	(82.9)	566	(26.1)		1.36 [0.96; 1.91]	
**Town size** #									.12		.044
< 20,000 inhabitants	14	(1.6)	141	(16.0)	726	(82.4)	881	(40.7)		Ref.	
[20,000 ; 100,000[ inhabitants	4	(1.4)	36	(12.6)	246	(86.0)	286	(13.2)		0.61 [0.40; 0.95]	
[100,000 ; 200,000[ inhabitants	6	(4.8)	23	(18.6)	95	(76.6)	124	(5.7)		1.46 [0.86; 2.50]	
≥ 200,000 inhabitants	18	(2.1)	127	(14.5)	731	(83.4)	876	(40.4)		0.92 [0.68; 1.24]	
**Region** #									.35		.18
Ile de France (includes Paris)	7	(1.7)	55	(13.2)	354	(85.1)	416	(19.2)		Ref.	
North-West	14	(2.9)	81	(16.6)	393	(80.5)	488	(22.5)		1.57 [1.03; 2.39]	
North-East	7	(1.4)	75	(14.4)	438	(84.2)	520	(24.0)		1.09 [0.72; 1.67]	
South-West	2	(0.9)	34	(15.3)	186	(83.8)	222	(10.3)		0.99 [0.58; 1.70]	
South-East	12	(2.3)	82	(15.7)	427	(82.0)	521	(24.0)		1.18 [0.78; 1.80]	
**Seasonal flu vaccination in the prior 3 years**									<.0001		<.0001
Never	10	(0.6)	188	(11.1)	1,500	(88.3)	1,698	(78.4)		Ref.	
Yes, at least once	32	(6.8)	139	(29.6)	298	(63.6)	469	(21.6)		3.21 [2.40; 4.29]	
**Personnally knows someone who contracted A/H1N1 flu**									.002		.006
No	27	(1.7)	238	(15.2)	1,302	(83.1)	1,567	(72.3)		Ref.	
Yes, in close environment (family, working colleagues)	13	(4.7)	51	(18.6)	210	(76.7)	274	(12.7)		1.65 [1.13; 2.41]	
Yes, outside close environment	2	(0.6)	38	(11.7)	286	(87.7)	326	(15.0)		0.75 [0.49; 1.13]	
**Belongs to priority groups for A/H1N1 vaccination**									<.0001		.002
No	23	(1.2)	263	(14.0)	1,595	(84.8)	1,881	(86.8)		Ref.	
Health care professionnal	13	(10.9)	11	(9.3)	95	(79.8)	119	(5.5)		0.86 [0.48; 1.52]	
Pregnant women	0	0	11	(37.9)	18	(62.1)	29	(1.3)		5.09 [1.86; 13.92]	
Other at-risk individuals with chronic diseases*	6	(4.4)	42	(30.4)	90	(65.2)	138	(6.4)		1.66 [1.05; 2.62]	
**Medical advice about A/H1N1 vaccination in the prior 6 months**									<.0001		<.0001
Did not have any medical consultation	5	(0.9)	84	(14.9)	474	(84.2)	563	(26.0)		Ref.	
Positive advice by a primary care physician	22	(12.7)	81	(46.8)	70	(40.5)	173	(8.0)		4.57 [2.92; 7.14]	
Positive advice by another health care professional	4	(7.8)	11	(21.6)	36	(70.6)	51	(2.3)		1.99 [0.94; 4.18]	
No positive advice by a health care professionnal	11	(0.8)	151	(10.9)	1,218	(88.3)	1,380	(63.7)		0.57 [0.42; 0.79]	
**Perception of severity of A/H1N1 influenza illness if infected**									<.0001		<.0001
Not at all severe or somewhat severe	14	(1.0)	129	(9.2)	1,254	(89.8)	1,397	(64.5)		Ref.	
Severe or very severe	28	(3.6)	198	(25.7)	544	(70.7)	770	(35.5)		3.61 [2.76; 4.71]	
**Self-perception of health state**									<.0001		NS
Poor or fair	11	(2.9)	78	(21.1)	281	(76.0)	370	(17.1)			
Good or very good or excellent	31	(1.7)	249	(13.9)	1,517	(84.4)	1,797	(82.9)			

*60 with asthma (43%); 19 with chronic bronchitis (14.7%); 46 with diabetes (33.3%); 13 with cardiac condition (9.4%).

†*chi^2^ test p-values (Cochran-Armitage test for age).*

‡*p-values for type III tests. Hosmer-Lemeshow test (p = 0.46) and deviance (p = 1.00) suggest that the goodness of fit was adequate for the final multivariate logistic model.*

#*covariates forced into multivariate model.*

*NS: Non significant after backward selection process, OR not provided.*

**Table 2:** Reasons for acceptability or not of A/H1N1 pandemic vaccination in the French adult population (online survey, November 17 to 25, 2009, N = 2,167).

Main reason(s) for acceptability of pandemic vaccination (n = 369)	% [CI 95%]
Protecting myself to avoid sickness	74.5 [69.8; 78.9]
Protecting my close relatives	68.8 [63.8; 73.5]
Getting vaccinated is convenient and quick	27.4 [22.9; 32.2]
A health professional advised me to get vaccinated	25.2 [20.9; 30.0]
Getting vaccinated is a civic duty	24.1 [19.8; 28.8]
Vaccination is recommended by public authorities	23.6 [19.3; 28.3]
Vaccination is free	21.1 [17.1; 25.7]
Protecting myself to avoid work absenteeism	20.1 [16.1;24.5]
Vaccines are safe	9.2 [6.5; 12.6]
Vaccines have no side effects	7.1 [4.7; 10.2]
**Main reason(s) for non-acceptability of pandemic vaccination (n = 1,798)**	
Vaccines are not safe enough	71.2 [69.0; 73.3]
Vaccines have side effects	68.4 [66.2; 70.1]
Flu is not a severe disease	19.7 [17.9; 21.7]
Vaccines lack efficacy	17.3 [15.6; 19.1]
A health professional advised me to avoid vaccination	15.3 [13.7; 17.0]
I never get the flu	15.0 [13.3; 16.7]
I dislike the shots	7.0 [5.8; 8.2]
Getting vaccinated is inconvenient and too long	3.6 [2.8; 4.6]
I have medical reasons to avoid H1N1 vaccine	1.4 [0.9; 2.1]

* Any items could be selected and thus proportions do not add to 100%. Items were presented in a random order.

Acceptability of A/H1N1 vaccination was significantly higher among pregnant women (37.9% -p = .003) and other at risk individuals with chronic diseases (34.8% -p<.001) as confirmed in multivariate analysis (Table 1). Acceptability of A/H1N1 vaccination was slightly higher among health care professionals (20.2%), but this difference was not statistically significant even in univariate analysis (p = .35). Among parents with children in the household (n = 1,030), 225 (21.8%) respondents were willing to get their children vaccinated; about a quarter (25.8%) of parents who accepted vaccination for their children did not accept vaccination for themselves. On the contrary, only 30 (3.7%) out of 805 parents who refused vaccination for their children did accept vaccination for themselves.

Table 1 also shows that acceptability of vaccination was significantly related to a number of socio-demographic characteristics, even after multivariate adjustment. Female respondents were less willing to get vaccinated than males (p = .0001). Acceptability of vaccination was significantly lower for adults less than 35 (p = .002) and increased with age (Cochran-Armitage test for trend: p<.0001). Respondents who graduated from high school or undergraduate studies at university were less willing to get vaccinated than others (p = .016). Clerks were less willing to get vaccinated than respondents with another social grade (p = .011). The presence of only one child in the household was associated with a higher acceptability when compared with both households with no child and those who had more than one child (p = .008). Acceptability of A/H1N1 vaccination was similar in all French regions but lower among respondents living in small towns with 20,000 to 100,000 inhabitants (p = .044).

At time of the survey, the majority of the French general population did not associate A/H1N1 influenza-pandemic with a serious threat. Only one third of respondents (35.5%) considered A/H1N1 influenza-pandemic illness as a “severe” or “very severe” disease (Table 1), and even less respondents declared that they were “worried” or “very worried” about the A/H1N1 influenza-pandemic for themselves, and that they personally felt at “higher risk than average” for contracting A/H1N1 influenza infection (15.1% and 8.2%, respectively). Respondents belonging to at-risk groups (including pregnant women) were more likely to be worried for themselves (23.9%) than others (14.4% -p<.01); in contrast, health care professionals were not more likely to be worried for themselves (15.1%) than others. A higher proportion of respondents (29.2%) expressed concerns (being “worried” or “very worried”) about the risk that one family member may contract A/H1N1 influenza infection, and this proportion was significantly higher among parents with children in the household (35.0% versus 23.8% in the rest of the sample -p<.001).

Respondents with a higher perception of the severity of influenza-pandemic illness were significantly more likely to accept vaccination, and this was confirmed after multivariate adjustment (Table 1; p<.0001). When alternative constructs of risk perceptions were introduced in the multivariate analysis, they also remain significant in each of the final models (p<.0001): being “worried” or “very worried” about the A/H1N1 influenza-pandemic for oneself (OR = 2.90; 95% CI: 2.12 to 3.97) or one's close relatives (OR = 3.38; 95% CI: 2.58 to 4.42); feeling personally at “higher risk than average” to contract A/H1N1 influenza infection (OR = 2.63; 95% CI: 1.75 to 3.85).

Respondents who had already been confronted to a case of A/H1N1 influenza-pandemic illness in their close relationships (family members and/or work colleagues) were more likely to accept A/H1N1 vaccination (Table 1; p = .006). It should be noted that acceptability of vaccination was lower among those who knew individuals who had contracted A/H1N1 influenza but not in their close relationships than among those who did not know any flu case (although this difference was not significant after multivariate adjustment).

Respondents who were vaccinated for seasonal influenza at least once in the prior three years were also more likely to accept A/H1N1 vaccination (Table 1; p<.0001). Similarly, less than half of respondents (46.1%) declared that they were always “compliant” with vaccinations recommended by their primary care physician, and acceptability of A/H1N1 pandemic vaccination was higher among them (25.9%) as compared to others (9.4%) (p<.0001). When introduced in the multivariate analysis instead of seasonal influenza immunization behavior, the level of compliance with vaccination remained significant in the final model (OR = 3.15; 95% CI: 2.39 to 4.15).

Nearly three quarters of the population (74.0%) had consulted a physician at least once in the prior 6 months (Table 1). Among these 1,604 respondents, 173 (8.0% of total sample) were formally advised by their primary care physician to get vaccinated, and 51 (2.3% of total sample) were advised to do so by another health care professional. About half (57/103) respondents being advised to get vaccinated by their primary care physician and accepting A/H1N1 vaccination declared that their physician's advice was their main motivation to do so. Among 167 respondents at risk for A/H1N1 influenza complications, 88.6% had at least one medical consultation in the prior six months, but only 25.0% were formally advised to get vaccinated. For 1,380 respondents (63.7% of total sample) who had at least one medical consultation, no physician took this opportunity to advise them to get A/H1N1 vaccination, while 232 respondents (10.7% of total sample) declared explicitly that A/H1N1 vaccination was discussed during the consultation and they were formally advised not to get vaccinated. Multivariate analysis confirmed that those who were not advised to get vaccinated by a health care professional were less likely to accept vaccination than those who did not have any medical consultation in the prior six months (p<.0001). On the other hand, it confirmed that a positive advice from a primary care physician significantly increased acceptability of vaccination; however, this was not confirmed in the case of a positive advice by other health care professionals.

Although the deviance of the final model suggested that main effects fit very well the data (deviance = 1,481 with DF = 2,106; p = 1.00), we looked for additional two-way interaction effects using a backward selection. Two interactions effects were retained that contrasted acceptability of A/H1N1 pandemic vaccination for risk perception and seasonal influenza immunization depending on the number of children in the household: 1) respondents who considered A/H1N1 influenza-pandemic illness as a “severe” or “very severe” disease had adjusted odds-ratios for acceptability of vaccination of 2.56 (CI 95%, 1.74 to 3.76) for those having no child; 3.74 (CI 95%, 2.21 to 6.32) for those having one child; and 6.59 (CI 95%, 3.84 to 11.32) for those having more than one child (p = .020); and 2) respondents who were vaccinated for seasonal influenza at least once in the prior three years had adjusted odds-ratios for acceptability of vaccination of 4.67 (CI 95%, 3.25 to 6.99) for those having no child; 2.85 (CI 95%, 1.61 to 5.04) for those having one child; and 1.69 (CI 95%, 0.93 to 3.06) for those having more than one child (p = .016). The main effects of the final model remained significant when the two interaction effects were added with exception of the number of children in the household (p = .15).

## Discussion

This cross-sectional survey took place from November 17 to 25, 2009, shortly after the mass vaccination campaign had started in the general population (November 12), i.e. twelve weeks after the influenza-pandemic occurred in France and a week before the peak (November 23–29 – Week 48) as surveillance epidemiological data revealed subsequently [18]. Overall, the acceptability of A/H1N1 vaccination was low at 17.0% (CI 95%, 15.5% to 18.7%) among 2,167 respondents representative of the French adult population aged 18 to 64. The majority of the French general population did not associate A/H1N1 influenza-pandemic illness with a serious threat. Only 35.5% of respondents perceived A/H1N1 influenza illness as a severe disease and 12.7% had experienced A/H1N1 cases in their close relationships with higher acceptability of vaccination (p<.0001 and p = .006, respectively). In comparison to 26.0% respondents who did not consult their primary care physician in the prior six months, acceptability was significantly higher among 8.0% respondents who were formally advised to get vaccinated by their primary care physician, and lower among 63.7% respondents who were not advised to get vaccinated (respectively: 15.8%, 59.5% and 11.7%- p<.0001). Among 1,798 respondents who refused vaccination, 71.2% expressed concerns about vaccine safety.

We found that risk perceptions of the A/H1N1 influenza-pandemic were strongly correlated to the acceptability of vaccination in the general population. It confirms findings from previous surveys conducted worldwide about attitudes and behaviors toward vaccination against seasonal flu [2], [16], the highly pathogenic A/H5N1 influenza virus [19], [20], [21], [22], and more recently the A/H1N1 influenza-pandemic [23], [24], [25], [26]. However, the majority of the French general population did not associate A/H1N1 influenza-pandemic with a serious threat, albeit a week before the pandemic peak [18].

The substantial impact of other determinants illustrates that while the perceived severity of the A/H1N1 influenza-pandemic may be a sufficient condition for getting vaccinated in a mass vaccination campaign, it is not a necessary one. We found that individual characteristics including male gender, older age, and previous receipt of seasonal influenza vaccine were independent predictors of the acceptability of A/H1N1 pandemic vaccination. The same individual characteristics were similarly associated with the acceptability of A/H1N1 pandemic vaccination in other countries [23], [24], [25], [26], but also with seasonal vaccine uptake in the whole French at-risk population aged less than 65 [3]. These findings suggest that prior beliefs and attitudes toward seasonal influenza vaccination are major leverages to increase the uptakes of influenza-pandemic vaccination in the general population.

However, we found that acceptability of A/H1N1 pandemic vaccination was as low as 17.0% among the French adult population, and concerns about A/H1N1 pandemic vaccine safety were the main reason quoted by 71.2% respondents who denied being vaccinated. In a Canadian qualitative study among health care professionals and the general public, the authors found that individuals were hesitant to accept pandemic vaccines and that “concerns about using new vaccines during a pandemic differ from concerns about using established products in non-crisis situations” [9]. For an emerging public health threat that diffuses very quickly, as it has been the case for the A/H1N1 influenza-pandemic, perceptions of the benefits and risks of vaccination may continuously evolve. Our results primarily suggest that the general population was not reassured that A/H1N1 pandemic vaccines were safe. It calls into question the information received by the general population at time of the survey, and what factors may have worsened the perception that A/H1N1 pandemic vaccines are unsafe.

At time of the survey, the A/H1N1 influenza-pandemic had attracted massive media coverage in France, albeit in two opposite directions. On the one hand, the severity of A/H1N1 2009 influenza illness was stressed by daily reports of fatalities in the news media (i.e. 357 hospitalizations in intensive care units (ICUs) and 68 deaths at time of the survey) [27], frequent messages from public health authorities, and personal appearances in the media of the Ministry of Health and the Head of State in order to motivate people's compliance with the mass vaccination campaign. On the other hand, the safety of A/H1N1 pandemic vaccines was scrutinized by the media with regard to the risk of Guillain-Barré syndrome, the limited knowledge about adjuvanted vaccines accounting for almost all doses available in France, the accelerated authorization procedure to market pandemic vaccines and the actual motivations of pharmaceutical firms, while the unclear number of vaccine injections called their protective efficacy into question.

Although the public's perception of a health risk usually increases with its coverage in the news media [28], this general trend may indeed be counteracted if this media information is dissonant [29], and if daily personal experience does not confirm the threat [30]. Previous population surveys in the US[31] and the UK[32] have emphasized that after an initially high level of risk perception, levels of anxiety waned along with the perception of the A/H1N1 influenza-pandemic as an immediate threat and that tackling the perception that the outbreak has been “over-hyped” may be difficult. In our study, 12.7% respondents reported a case of A/H1N1 flu in their close environment. The fact that these respondents had a significantly higher acceptability of A/H1N1 pandemic vaccination suggests that they saw the A/H1N1 influenza-pandemic as a real threat in concordance with messages from public health authorities. On the contrary, dissonance may have grown in the vast majority of the general population who had no (72.3%) or indirect (15.0%) experience with A/H1N1 influenza-pandemic.

As a consequence, 74% respondents looked for medical advice about A/H1N1 pandemic vaccination, an estimate above the expected number of consultations for a similar period [15]. Previous studies have shown that behaviors, attitudes, and advice from primary care physicians were strongly associated with their patients' immunization behavior for seasonal influenza [2], [11], [12], [13] as well as the “swine flu” in the 1976 mass vaccination campaign in the U.S. [33]. We found consistently that a positive advice from a primary care physician was a major determinant of the acceptability of A/H1N1 pandemic vaccination. However, 63.7% respondents were not advised to get vaccinated with significantly lower acceptability of A/H1N1 pandemic vaccination. First and foremost, this finding is in accordance with the low uptake rate (10.9%) reported at time of the survey for health care professionals who were the first priority group to access pandemic vaccines [5], [34]. To the extent that 62% of general practitioners were willing to get vaccinated during the summer of 2009 [35], future studies should explore whether their behaviors and attitudes toward A/H1N1 pandemic vaccines did evolve negatively as a result of risk communication of public health authorities and/or their dismissal from the mass vaccination campaign decided on August 21, 2009 [4]. Assumingly, the decision to strictly administer A/H1N1 pandemic vaccines in ad hoc vaccination centers had further increased dissonance in the general population since it was in sharp contrast with the past experience of the general population learnt from seasonal flu vaccination that is mainly prescribed and administered by primary care physicians in France [3], as well as policies adopted by neighboring countries, like Belgium, Germany, and the UK [36].

Finally, we found that parents had a higher acceptability of A/H1N1 pandemic vaccination for themselves than other adults without children. Further analysis showed that such higher acceptability was mediated by the perception of A/H1N1 influenza-pandemic illness as a “severe” or “very severe” disease with an increased acceptability depending on the number of children in the household. However, parents who denied vaccination for themselves expressed significantly more concerns about vaccine safety than other adults without children (76.5% vs. 66.6%, respectively; p<.0001). Quite logically, parents were reluctant to get their children vaccinated; only a quarter of parents accepted vaccination for their children but not for themselves. Future studies should address more specifically knowledge, attitudes and behaviors of parents about pandemic vaccination of their children since children are the most important drivers of influenza infection and may be targeted for pandemic vaccination before their parents [5].

Our study is subject to a number of weaknesses. The advantage of our Web-based sampling strategy is the ability to quickly deploy a survey and thereby track responses in near real-time knowing that risk perceptions and attitudes toward pandemic vaccination may continuously evolve [23], [31]. The possible disadvantage of this strategy is a sacrifice of population representativeness. A non-coverage bias is limited by the quite high Internet coverage in the French adult population (estimated at 67% in 2008), while coverage rates are the highest in our target population of adults aged 18 to 64 [37]. The representativeness of online data collection is also established to the extent that it follows the procedure used in this survey, i.e. stratified random sampling in a pre-existing large representative panel of the whole population [38], [39].

Although we cannot unequivocally rule out the existence of selection bias in our online sample, our analyses are consistent with the view that our sample is representative of the French adult population aged 18 to 64 as compared to previous surveys conducted in random samples with use of traditional methods for data collection (face to face or phone interviews): 1) random sampling in our survey was stratified to match French official census statistics for gender, age, occupation, household size, size of the population in the area of residence, and region [17]; 2) 22% of respondents received seasonal influenza vaccination at least once in the prior three years consistent with national uptake rates around 22–24% over recent years [2]; and 3) usual explanatory factors for seasonal flu uptake were also consistently found to associate with acceptability of A/H1N1 vaccination (i.e. male gender, older age, previous vaccination against seasonal flu, groups at risk for influenza complications) [2], [3].

Although such online survey shares with other survey methods the general limitations of results based on respondent's self-declarations, it is well established that self-administered questionnaires tend to yield fewer reports in the socially desirable direction than do interviewer-administered questionnaires, and a recent study suggested that online surveys may have the lowest social desirability bias [40]. In particular, the validity of our results was further supported by actual immunization behaviors reported in official statistics: the low uptake of A/H1N1 vaccine among health care professionals (10.7%; CI 95%: 5.8% to 17.7%), who were the first priority group to access vaccines on October 20, 2009, was similar to the actual uptake rate (10.9%) reported at time of the survey [34]; the low acceptability of A/H1N1 vaccination in the French general population (17.0%; CI 95%, 15.5% to 18.7%) predicted the low coverage rate (2.7 million people, i.e. 7.1% coverage in the population aged 18 to 60) reported on February 28, 2010 [41].

The uptake of A/H1N1 pandemic vaccines appears to be very low in France as compared to some other European Union and North American countries that have undertaken a mass vaccination campaign [42], [43]. While risk perceptions of A/H1N1 influenza-pandemic were expectedly found to drive immunization behaviors, the majority of the adult population expressed concerns about pandemic vaccines' safety and refused vaccination for themselves and their children. As evidence by this study and others, risk communication of public health authorities should primarily focus on reassuring the general population that pandemic vaccines are safe [41]. In addition, our study shows that the implementation of a mass vaccination campaign and the particular role given to primary care physicians were major factors to achieve a successful pandemic vaccination campaign. On January 11, 2010, the French Ministry of Health reversed its policy and authorized primary care physicians to administer A/H1N1 vaccines. While such policy change should contribute to increase significantly uptake rates among priority groups at risk for A/H1N1 influenza-pandemic complications, it may have occurred too late to change uptake rates in the general population at a time the fist wave of the influenza-pandemic ended [44].

## References

[1] World Health Organization (2009). http://www.who.int/csr/disease/swineflu/notes/h1n1_vaccine_20090713/en/index.html.

[2] Blank PR, Schwenkglenks M, Szucs TD (2008). Influenza vaccination coverage rates in five European countries during season 2006/07 and trends over six consecutive seasons.. BMC Public Health.

[3] Tuppin P, Samson S, Weill A, Ricordeau P, Allemand H (2009). [Influenza vaccination coverage in France in 2007-2008: contribution of vaccination refund data from the general health insurance scheme].. Med Mal Infect.

[4] Ministère de l'Intérieur, Ministère de la Santé et des Sports (2009). http://www.sante-sports.gouv.fr/IMG/pdf/Circulaire_vaccination_090824.pdf.

[5] Haut Conseil de la Santé Publique (2009). http://www.hcsp.fr/docspdf/avisrapports/hcspa20090907_H1N1.pdf.

[6] Brewer NT, Chapman GB, Gibbons FX, Gerrard M, McCaul KD (2007). Meta-analysis of the relationship between risk perception and health behavior: the example of vaccination.. Health Psychol.

[7] Leppin A, Aro AR (2009). Risk perceptions related to SARS and avian influenza: theoretical foundations of current empirical research.. Int J Behav Med.

[8] Sencer DJ, Millar JD (2006). Reflections on the 1976 swine flu vaccination program.. Emerg Infect Dis.

[9] Henrich N, Holmes BJ (2009). The public's acceptance of novel vaccines during a pandemic: A focus group study and its application to influenza H1N1.. Emerging Health Threats Journal.

[10] Black S, Eskola J, Siegrist CA, Halsey N, Macdonald N (2009). Importance of background rates of disease in assessment of vaccine safety during mass immunisation with pandemic H1N1 influenza vaccines.. Lancet.

[11] Frank E, Rothenberg R, Lewis C, Belodoff BF (2000). Correlates of physicians' prevention-related practices. Findings from the Women Physicians' Health Study.. Arch Fam Med.

[12] Nichol KL, Zimmerman R (2001). Generalist and subspecialist physicians' knowledge, attitudes, and practices regarding influenza and pneumococcal vaccinations for elderly and other high-risk patients: a nationwide survey.. Arch Intern Med.

[13] Maurer J (2009). Who has a clue to preventing the flu? Unravelling supply and demand effects on the take-up of influenza vaccinations.. J Health Econ.

[14] Eysenbach G (2004). Improving the quality of Web surveys: the Checklist for Reporting Results of Internet E-Surveys (CHERRIES).. J Med Internet Res.

[15] Lanoe J, Makdessi-Raynaud Y (2005). http://www.sante.gouv.fr/drees/etude-resultat/er436/er436.pdf.

[16] Hollmeyer HG, Hayden F, Poland G, Buchholz U (2009). Influenza vaccination of health care workers in hospitals-A review of studies on attitudes and predictors.. Vaccine.

[17] Institut National de la Statistique et des Etudes Economiques (2006). http://www.recensement.insee.fr/home.action.

[18] Institut de Veille Sanitaire (2010). http://www.invs.sante.fr/surveillance/grippe_dossier/default.htm.

[19] Lau JT, Kim JH, Tsui H, Griffiths S (2007). Perceptions related to human avian influenza and their associations with anticipated psychological and behavioral responses at the onset of outbreak in the Hong Kong Chinese general population.. Am J Infect Control.

[20] Lau JT, Kim JH, Tsui HY, Griffiths S (2008). Perceptions related to bird-to-human avian influenza, influenza vaccination, and use of face mask.. Infection.

[21] Barr M, Raphael B, Taylor M, Stevens G, Jorm L (2008). Pandemic influenza in Australia: using telephone surveys to measure perceptions of threat and willingness to comply.. BMC Infect Dis.

[22] Di Giuseppe G, Abbate R, Albano L, Marinelli P, Angelillo IF (2008). A survey of knowledge, attitudes and practices towards avian influenza in an adult population of Italy.. BMC Infect Dis.

[23] Quinn SC, Kumar S, Freimuth VS, Kidwell K, Musa D (2009). Public willingness to take a vaccine or drug under Emergency Use Authorization during the 2009 H1N1 pandemic.. Biosecur Bioterror.

[24] Sypsa V, Livanios T, Psichogiou M, Malliori M, Tsiodras S (2009). Public perceptions in relation to intention to receive pandemic influenza vaccination in a random population sample: evidence from a cross-sectional telephone survey.. Euro Surveill.

[25] Lau JT, Yeung NC, Choi KC, Cheng MY, Tsui HY (2009). Acceptability of A/H1N1 vaccination during pandemic phase of influenza A/H1N1 in Hong Kong: population based cross sectional survey.. BMJ.

[26] Eastwood K, Durrheim DN, Jones A, Butler M (2010). Acceptance of pandemic (H1N1) 2009 influenza vaccination by the Australian public.. Med J Aust.

[27] Institut de Veille Sanitaire (2009). http://www.invs.sante.fr/surveillance/grippe_dossier/default.htm.

[28] Breakwell G (2007). The Psychology of Risk..

[29] Young ME, Norman GR, Humphreys KR (2008). Medicine in the popular press: the influence of the media on perceptions of disease.. PLoS One.

[30] Voeten HA, de Zwart O, Veldhuijzen IK, Yuen C, Jiang X (2009). Sources of information and health beliefs related to SARS and avian influenza among Chinese communities in the United Kingdom and The Netherlands, compared to the general population in these countries.. Int J Behav Med.

[31] Jones JH, Salathe M (2009). Early assessment of anxiety and behavioral response to novel swine-origin influenza A(H1N1).. PLoS One.

[32] Rubin GJ, Amlot R, Page L, Wessely S (2009). Public perceptions, anxiety, and behaviour change in relation to the swine flu outbreak: cross sectional telephone survey.. BMJ.

[33] Cummings KM, Jette AM, Brock BM, Haefner DP (1979). Psychosocial determinants of immunization behavior in a swine influenza campaign.. Med Care.

[34] Ministère de l'Intérieur (2009). http://www.interieur.gouv.fr/sections/a_la_une/toute_l_actualite/grippea-h1n1/point-campagne-vaccination.

[35] Schwarzinger M, Verger P, Guerville MA, Aubry C, Rolland S (2010). Positive attitudes of French general practitioners towards A/H1N1 influenza-pandemic vaccination: A missed opportunity to increase vaccination uptakes in the general public?. Vaccine.

[36] Kmietowicz Z (2009). GPs are to be paid £5.25 a shot in the swine flu vaccination programme.. BMJ.

[37] Bigot R, Croutte P (2008). http://www.credoc.fr/publications/abstract.php?ref=R256.

[38] Fielding N, Lee R, Blank G (2008). The SAGE Handbook of Online Research Methods..

[39] Baker L, Wagner TH, Singer S, Bundorf MK (2003). Use of the Internet and e-mail for health care information: results from a national survey.. JAMA.

[40] Kreuter F, Presser S, Tourangeau R (2008). Social desirability bias in CATI, IVR, and Web surveys. The effects of mode and question sensitivity.. Public Opin Q.

[41] Agence Française de Securité Sanitaire des Produits en Santé (2010). http://www.afssaps.fr/Dossiers-thematiques/Pandemie-grippale/Surveillance-des-effets-indesirables-des-antiviraux-et-des-vaccins/%28offset%29/3.

[42] De nos correspondants (2010). http://www.lemonde.fr/epidemie-grippe-a/article/2010/01/05/avec-60-de-sa-population-vaccinee-la-suede-figure-loin-devant-la-plupart-des-pays_1287619_1225408.html.

[43] MMWR Morb Mortal Wkly Rep.

[44] Chowell G, Viboud C, Wang X, Bertozzi SM, Miller MA (2009). Adaptive vaccination strategies to mitigate pandemic influenza: Mexico as a case study.. PLoS One.

